# Comparison of water-use characteristics of tropical tree saplings with implications for forest restoration

**DOI:** 10.1038/s41598-021-81334-0

**Published:** 2021-01-18

**Authors:** Tushar Andriyas, Nisa Leksungnoen, Pantana Tor-ngern

**Affiliations:** 1 Graduate School, Chulalongkorn University, Bangkok, 10330 Thailand; 2 Department of Forest Biology, Faculty of Forestry, Kasetsart University, Bangkok, 10900 Thailand; 3 Department of Environmental Science, Faculty of Science, Chulalongkorn University, Bangkok, 10330 Thailand; 4 Environment, Health and Social Data Analytics Research Group, Chulalongkorn University, Bangkok, 10330 Thailand; 5 Water Science and Technology for Sustainable Environment Research Group, Chulalongkorn University, Bangkok, 10330 Thailand

**Keywords:** Environmental sciences, Ecophysiology

## Abstract

Tropical forests are experiencing reduced productivity and will need restoration with suitable species. Knowledge of species-specific responses to changing environments during early stage can help identify the appropriate species for sustainable planting. Hence, we investigated the variability in whole-tree canopy conductance and transpiration (*G*_*t*_ and *E*_*L*_) in potted saplings of common urban species in Thailand, viz., *Pterocarpus indicus*, *Lagerstroemia speciosa*, and *Swietenia macrophylla*, across wet and dry seasons in 2017–2018. Using a Bayesian modeling framework, *G*_*t*_ and *E*_*L*_ were estimated from sap flux density, informed by the soil, atmospheric and tree measurements. Subsequently, we evaluated their variations with changing vapor pressure deficit (VPD) and soil moisture across timescales and season. We found that *G*_*t*_ and *E*_*L*_ were higher and highly variable in *L. speciosa* across seasons than *S. macrophylla* and *P. indicus*. Our results implied that water-use in these species was sensitive to seasonal VPD. *L. speciosa* may be suitable under future climate variability, given its higher *G*_*t*_ and *E*_*L*_ across atmospheric and soil moisture conditions. With their lower *G*_*t*_ and *E*_*L*_, *P. indicus* and *S. macrophylla* may photosynthesize throughout the year, maintaining their stomatal opening even under high VPD. These findings benefit reforestation and reclamation programs of degraded lands.

## Introduction

Tropical forests are one of the largest carbon sinks in terrestrial ecosystems^1^. Climate change has been predicted to result in more intense with protracted dry or wet spells, with varying duration, intensity, frequency, and spatiotemporal spread in different parts of the world^2,3^. In fact, drought-induced mortality of global tropical forests has been recently documented^4^. Species mortality caused by anthropogenic factors can be important^5^ and can result in a loss of carbon storage, leading to a cyclical downturn of increasing temperatures and further mortality. Therefore, many countries are taking preemptive action to reduce such impacts through reforestation. Reforestation includes planting trees on previously degraded lands, which can be demanding on newly planted trees. Under the predicted intensification of climate change, reforested trees may experience harsh conditions, resulting in an unsuccessful establishment. To ensure successful establishment in degraded lands, species robustness to seasonal variations, especially under a climate change scenario is needed. Because trees in urban conditions likely experience extreme environmental stresses from heat, drought, and flood, another interesting approach is to study the tolerance and resilience of urban tree species to climate variability which will infer the physiological responses of trees and their vulnerability to climate extremes^5,6^.

In Bangkok, street tree species differ in leaf phenology (i.e., deciduous or evergreen), habitat (ranging from hill evergreen forest to mangrove forest), and origin (native or exotic)^7^, with the most common street tree species being *Pterocarpus indicus*, *Lagerstroemia speciosa*, and *Swietenia macrophylla*^8^. *P. indicus* is a fast-growing medium-size tree native to Asia and can be either deciduous or facultative evergreen, depending on the availability of soil moisture and the openness of the growth area. *L. speciosa* is a fast-growing medium-size tree native to Southeast Asia and is a deciduous species, usually found in mixed deciduous forests. *S. macrophylla* is an evergreen species native to South and Central America and was introduced into Thailand due to its fast growth and adaptability to various environmental conditions.

The robustness of a given tree species for forest establishment is inferred by investigating its physiological responses to variations in atmospheric demand (i.e., seasonal variations) and soil water availability, especially during the early stages of growth. Variability in soil water availability and atmospheric demand make it essential to estimate the variations in plant ecohydrological process/processes^9^. Some relevant variables for estimating such responses are transpiration and photosynthesis, which represent water use and carbon gain of trees. Many investigations have been widely conducted in trees of various growing stages by measuring leaf level transpiration and photosynthesis^10–13^. However, during leaf-level experiments, it can be difficult to monitor the continual changes in variables, limiting the amount of useful information that can be gained by estimating such responses. Moreover, physiological responses at leaf scale may not directly translate to those at the canopy scale^14–17^. An alternative approach is to analyze the whole-tree transpiration and photosynthesis in response to changing environmental conditions. However, whole-tree photosynthesis is usually difficult to estimate without detailed measurements of biochemical photosynthetic parameters and leaf nutrients, as required in most photosynthesis models^18,19^. Instead, canopy conductance may be used to infer a tree’s ability in capturing the atmospheric carbon dioxide and to impute the responses of whole-tree photosynthesis to environmental changes.

Common techniques for estimating such parameters include sap flux probes which continuously detect changes in water flow rates in stems and are of various designs (Heat dissipation^20,21^, Heat pulse^22^, Heat ratio^23^, Heat balance^24^). Data from sap flux probes can be scaled from a point measurement in the stem to whole-tree transpiration and canopy conductance^25–28^. As such, proper scaling is needed to estimate the canopy conductance^29,30^, transpiration^31^, and carbon assimilation^32^. However, there are inherent errors and uncertainties involved in the scaling process. Additionally, errors can result from failing to account for capacitance in stems, which causes a time lag between both fluxes and atmospheric evaporative demand^33,34^. Another issue is missing data which come from unexpected sensor failure. This can result in an inability to obtain a continuous data set for analyses across temporal scales.

Normally, gap filling of missing data is performed by applying relationships between the measured sap flux data and environmental factors such as vapor pressure deficit (VPD) and soil moisture^35^. However, such an approach may not capture the true physiological responses of trees and is unable to recover continuous data when the environmental data are also missing. Therefore, a coherent probabilistic specification is needed to account for uncertainty resulting from sensor failure^36^, as unusable data points can cause scaling errors (from sap flux measurements to canopy processes)^37,38^. To overcome these limitations, a method based on hierarchical Bayesian statistics, called State-Space Canopy Conductance (StaCC) model, was developed recently^31^. The StaCC model uses sap flux density (point observation in stems) to infer transpiration and canopy conductance (at whole-tree or canopy scale) by explicitly accounting for the above uncertainties associated with the internal tree hydraulics and measurement errors occurred during observation. While estimating the canopy transpiration and conductance, the model also allows simultaneous gap-filling of missing sap flux data based on prior inference on canopy processes. The model substantially improves the estimation of canopy parameters compared to traditional scaling methods^31^.

With this context, we estimated whole-tree canopy conductance (*G*_*t*_) and transpiration (*E*_*L*_) of saplings of three common street tree species in Bangkok, Thailand, and evaluated their variations with atmospheric and soil moisture, with implications for potential establishment in degraded lands. The specific aims were to (1) determine the seasonal and interspecific difference in *G*_*t*_ and *E*_*L*_ in potted saplings of *P. indicus*, *L. speciosa*, and *S. macrophylla* (2) assess the extent to which such differences are a response to soil moisture and atmospheric evaporative demand (i.e., VPD). Additionally, we performed analysis across temporal scales, ranging from daily to monthly, to gain insights into the difference in physiological responses among the species, if exist, and whether it preserves across timescales. To estimate *G*_*t*_ and *E*_*L*_, a joint specification of tree-level (sap flux density, sapwood area, and leaf area) and environmental (air temperature, VPD, and soil moisture) data, together with model uncertainty was used to build an inference^36^. We employed the StaCC model on sap flux density data that were monitored on these saplings for 6 months (from 25th July 2017 to 11th February 2018), covering parts of a dry and a wet season. Findings from this study will benefit the selection of tree species for reforestation of lands, formerly under agriculture and logged forests, which can sometimes be found adjacent to national parks in Thailand. Such areas may be preserved to act as a buffer zone between the protected area and community lands.

## Results

### Model performance

An initial sensitivity analysis was done to determine the capacitance parameter, *β* (i.e., *β* was varied between 0.22 and 1 or a storage time between 120 to 0 min), as indicated by high R^2^ and significant *p* values. The *β* parameter represents the water storage capacity in stems which can be discharged through transpiration, buffering its daily fluctuations. In the context of our analysis, the response in sap flux density can be dampened by lags in stomatal response and capacitance (i.e., water storage). Additionally, we also ensured that model parameters converged to clear posterior distributions. These were obtained at different values of *β* for the three species, during a given season. Additionally, based on our analysis, keeping the prior on *β* weak (i.e., non-informative prior) improved the model predictions further. Hence, for a given species and season, we initialized the simulation with a given *β* value, which stabilized to a final value within the first 1000 iterations. Table 1 indicates the values with which the simulation was initialized (as determined through the sensitivity analysis) and the mean value obtained after removing the burn-in. For *P. indicus* and *L. speciosa* during the wet season, the variations in sap flux density were best explained by storage time which was around half of that during the dry season, while *S. macrophylla* had a higher storage time in the wet season. A lower storage time means that the lag between the stored water and the transpirational water is short, causing a relatively fast response to low soil moisture.

**Table 1:** Initial and mean values of the species capacitance parameter during wet and dry seasons.

Species	*β* Wet [initial, mean]	*β* Dry [initial, mean]	Mean storage time, wet, dry [Mins]
*P. indicus*	0.63, 0.83	0.63, 0.63	17, 30
*L. speciosa*	0.63, 0.81	0.86, 0.65	18, 29
*S. macrophylla*	0.86, 0.72	0.86, 0.86	23, 15

The last column indicates the storage time in minutes based on the mean *β* value.

### Prediction of sap flux density

**Figure 1:**
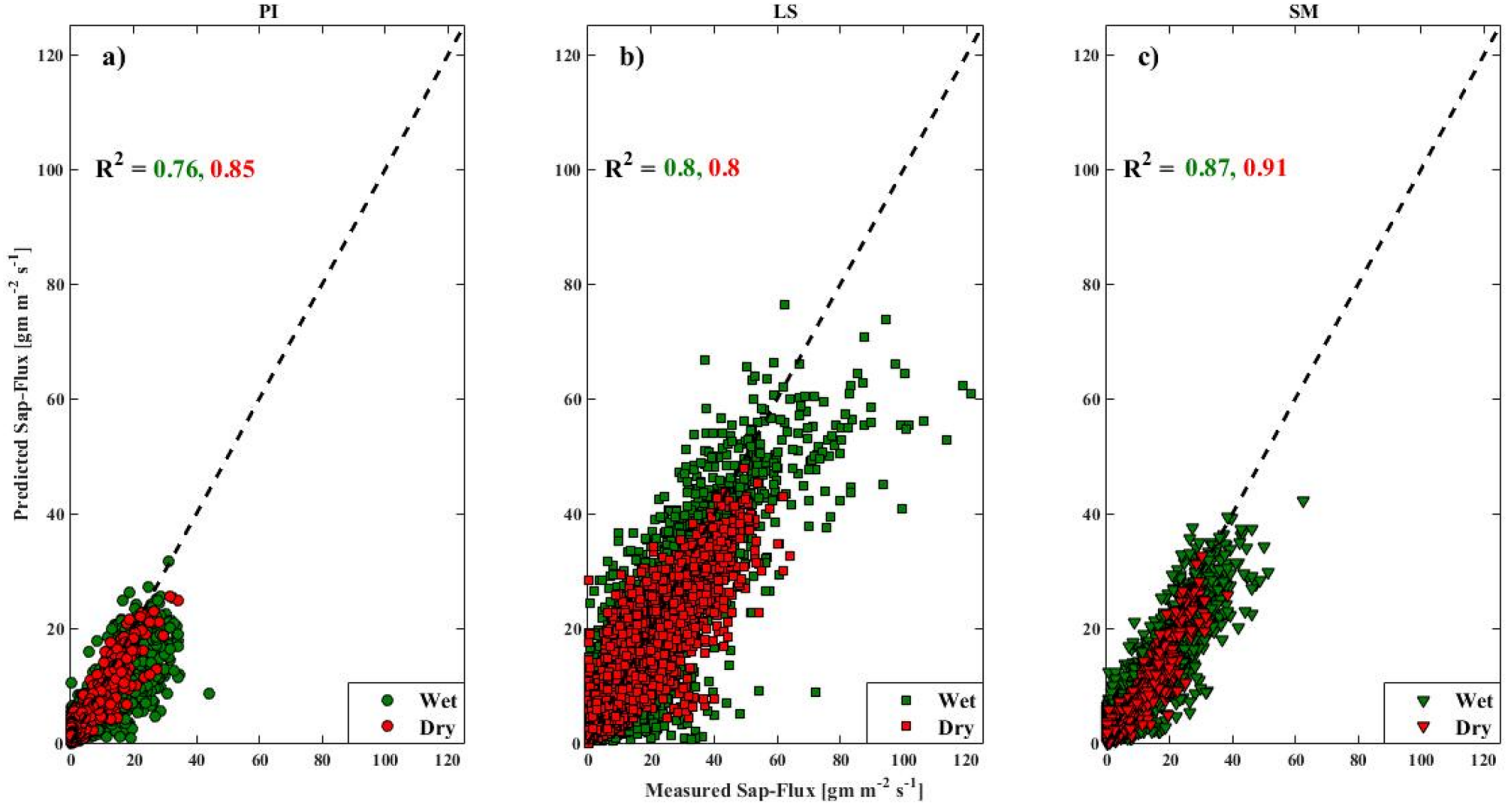
The best model of sap flux density prediction for (**a**) *Pterocarpus indicus* (PI), (**b**) *Lagerstroemia speciosa* (LS), and (**c**) *Swietenia macrophylla* (SM). The green (red) filled circles represent sap flux density data during the wet (dry) season. The corresponding R^2^ value for each season is also indicated. The black dashed line is the 1:1 line. This figure was generated using MATLAB version 7.12.0 R2017a (https://www.mathworks.com/products/new_products/release2017a.html).

The model performance varied with species and seasons, with predictions being the closest for *S. macrophylla*, intermediate in *P. indicus*, and the lowest for *L. speciosa*. Also, the model performance was relatively better during the wet season. The R^2^ values of the relationship between modeled and measured sap flux density during the wet (dry) season ranged 0.44–0.76 (0.40–0.85) for *P. indicus*, 0.46–0.80 (0.39–0.80) for *L. speciosa*, and 0.75–0.87 (0.61–0.91) for *S. macrophylla*, while the corresponding mean R^2^ values were 0.64 (0.65) for *P. indicus*, 0.70 (0.57) for *L. speciosa*, and 0.81 (0.78) for *S. macrophylla*. The corresponding average root mean squared error (RMSE) was 2.73 (2.10), 4.00 (2.71), and 3.36 (2.57) g m^−2^ s^−1^ for *P. indicus*, *L. speciosa*, and *S. macrophylla*, respectively. The residuals, which indicate the difference between predicted and measured sap flux values, did not deviate appreciably from a normal distribution. The scatter plots of the best sap flux density’s predictions (as indicated by the highest R^2^ value) are plotted in Fig. 1. Around the highest 1 to 3.5% daytime and the lowest nighttime, sap flux values, on average, were under and overestimated by the model, respectively. This prediction behavior may be associated with the unused PAR sub-model, but this did not influence our results as previously explained about the indifference between model runs with and without the PAR sub-model. On average, the model was able to capture the diurnal trends in sap flux density with the residuals not having an inherent bias.

### Canopy conductance and transpiration

**Figure 2:**
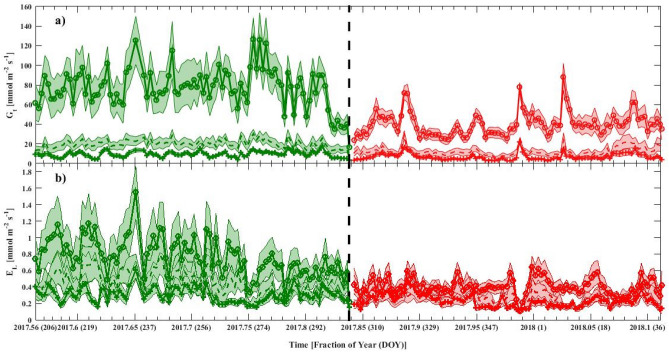
Estimated daily averaged (**a**) canopy conductance (*G*_*t*_) and (**b**) leaf transpiration (*E*_*L*_) during the wet (green) and dry (red) season. The vertical dash line indicates the ending of the wet season (DOY 305 or November 1st, 2017). Plots for *P. indicus* are depicted in + −, *L. speciosa* in o-, and *S. macrophylla* in—markers, respectively. The shaded regions represent the 95% confidence intervals. This figure was generated using MATLAB version 7.12.0 R2017a (https://www.mathworks.com/products/new_products/release2017a.html).

Sap flux measurements indicate that water consumption is different among the species and seasons. We observed a large degree of variation in seasonal and interspecific magnitude of reference conductance (*G*_*ref*_) and relative stomatal sensitivity to VPD was observed (figure not shown). *P. indicus* had similar *G*_*ref*_ in both seasons, but was highly sensitive to VPD during the dry season. As indicated in Table 2, whole-tree canopy conductance varied the greatest in *L. speciosa* with the relative sensitivity to natural log of VPD being low (λ/*G*_*ref*_ < 0.50) during both seasons for all the species, with only exception being *P. indicus* during the dry season (λ/*G*_*ref*_ > 0.70). Both *G*_*t*_ and *E*_*L*_ showed similar seasonal variation for a given species but with varying intensity (Fig. 2). The uncertainty in daily *G*_*t*_ and *E*_*L*_ averages was high during the wet season, indicating that the variability in VPD and soil moisture contributed greatly, relative to the model error. Such errors can be amplified further at much shorter, half-hourly timescale, as indicated by the variable R^2^ for the three species (Fig. 1).

**Table 2:** Mean stomatal sensitivity along with mean daily, weekly and monthly *G*_*t*_ and *E*_*L*_ (± SD) for the three investigated species during the wet and the dry season.

Species	Season	Daily *G*_*t*_ (mmol m^−2^ s^−1^)	Weekly *G*_*t*_ (mmol m^−2^ s^−1^)	Monthly *G*_*t*_ (mmol m^−2^ s^−1^)	Daily *E*_*L*_ (mmol m^−2^ s^−1^)	Weekly *E*_*L*_ (mmol m^−2^ s^−1^)	Monthly *E*_*L*_ (mmol m^−2^ s^−1^)
*P. indicus*	Wet	9.4 ± 3^E^	9 ± 2.3^C^	8.9 ± 1.8^C^	0.30 ± 0.1^C^	0.29 ± 0.1^C^	0..31 ± 0.03^C^
	Dry	6.7 ± 3.4^F^	6.6 ± 2.5^C^	6.8 ± 0.9^C^	0.23 ± 0.1^D^	0.22 ± 0.1^C^	0.23 ± 0.04^C^
*L. speciosa*	Wet	77.1 ± 19.6^A^	73.6 ± 20.1^A^	71.6 ± 18^A^	0.76 ± 0.2^A^	0.73 ± 0.2^A^	0.73 ± 0.2^A^
	Dry	40.2 ± 11.9^B^	39.9 ± 8.04^A^	40.5 ± 2.4^A^	0.40 ± 0.1^B^	0.40 ± 0.1^A^	0.41 ± 0.02^A^
*S. macrophylla*	Wet	20 ± 4.3^C^	19.2 ± 4.5^B^	19.1 ± 3.1^B^	0.44 ± 0.1^B^	0.43 ± 0.1^B^	0.45 ± 0.1^B^
	Dry	12.4 ± 4.3^D^	12.4 ± 3^B^	12.9 ± 1.8^B^	0.28 ± 0.1^C^	0.28 ± 0.1^B^	0.29 ± 0.04^B^
Species		< 0.0001	< 0.0001**	< 0.0001**	< 0.0001	< 0.0001**	< 0.0001**
Seasons		< 0.0001	< 0.0001**	< 0.0001**	< 0.0001	< 0.0001**	< 0.0001**
Species × seasons		< 0.0001**	0.409	0.334	< 0.0001**	0.202	0.230
Remarks			Wet > dry	Wet > dry		Wet > dry	Wet > dry

The same alphabetical superscript indicates no significant difference from each other and vice versa at 5% significant level.

*P*-values in bold and having ** indicate a statistically significant difference between means of the main factors and the interaction between the main factors.

Overall, the mean values of both *G*_*t*_ and *E*_*L*_ were statistically different among species and between seasons. *L. speciosa* had the highest *G*_*t*_ and *E*_*L*_ while *P. indicus* had the lowest values. Both *G*_*t*_ and *E*_*L*_ were significantly higher in wet season than in dry season. This difference was more pronounced in *G*_*t*_, where, for *L. speciosa*, it was about 10 times higher than *P. indicus,* while *E*_*L*_ was approximately 2 times higher. However, only the mean daily values indicated a significant interaction between species and seasons. This may be attributed to the lower number of samples in the weekly and monthly analysis, leading to a large variation and undetected difference in the ANOVA test.

Daily, weekly, and monthly *G*_*t*_ and *E*_*L*_ were the greatest in *L. speciosa* in either of the season and were the lowest for *P. indicus* (Table 2). The mean *G*_*t*_ and *E*_*L*_ for *L. speciosa* during the wet season was around 77 mmol m^−2^ s^−1^ and 0.76 mmol m^−2^ s^−1^, respectively and reached their maximum value (around 140 mmol m^-2^ s^-1^ and 1.6 mmol m^−2^ s^−1^, respectively) around mid-July, 2017. This can be attributed to the low total leaf area of *L. speciosa* (averaged 2.55 m^2^) compared to *P. indicus* and *S. macrophylla*, which had similar leaf areas (averaged ~ 3 m^2^). In general, *G*_*t*_ declined drastically for *L. speciosa*, with the other two species experiencing marginal declines as time progressed from wet to dry season, till the end of the study period.

### Diurnal variations

**Figure 3:**
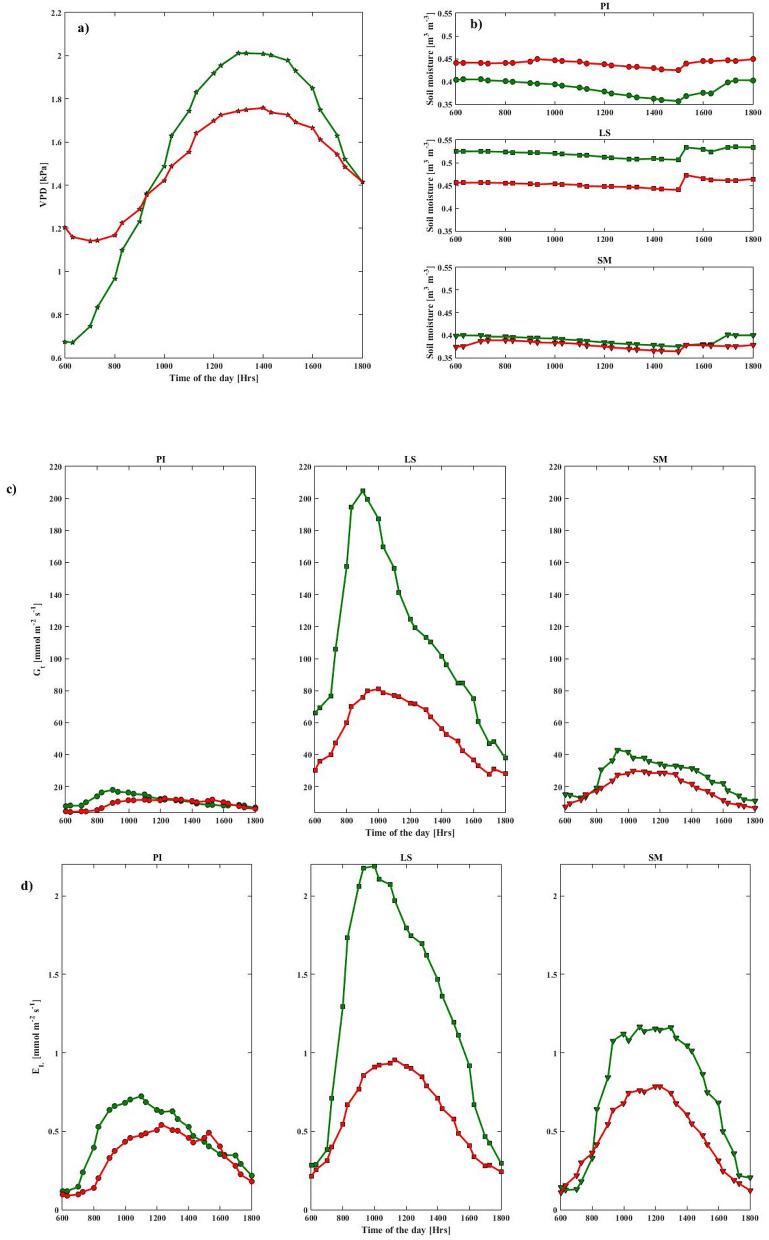
Hourly variations in (**a**) vapor pressure deficit (VPD; kPa), (**b**) soil moisture (m^3^ m^−3^), (**c**) canopy conductance (*G*_*t*_; mmol m^−2^ s^−1^) and (**d**) whole-tree transpiration (*E*_*L*_; mmol m^−2^ s^−1^), averaged over 6 AM to 6 PM LT of all days in the study period, during the dry and wet season. Columns correspond to the three species (*P. indicus* (PI); leftmost panel, *L. speciosa* (LS); middle panel, and *S. macrophylla* (SM); right panel) and seasons plotted in green (wet) and red (dry) symbols. This figure was generated using MATLAB version 7.12.0 R2017a (https://www.mathworks.com/products/new_products/release2017a.html).

During both seasons, VPD variations over the day were between 0.7 and 2.0 kPa, with VPD being significantly higher during the wet season than in the dry season from 8:00 to 17:00 local time (LT) (Fig. 3a). The soil moisture content measured in each species was lower during the dry season (Fig. 3b). The course of hourly *G*_*t*_ and *E*_*L*_ (averages from 6:00 to 18:00 LT) are plotted for the three species (Fig. 3c and d, respectively). A uni-modal pattern was observed in both *G*_*t*_ and *E*_*L*_ in the three species, showing peaks between 8:00 and 14:00 LT (Fig. 3c and d). Maximum *G*_*t*_ was reached early in the day (around 9:00 LT) for *L. speciosa,* while the *E*_*L*_ rates peaked approximately 2 h later during the wet season. A sudden rise to high values during the early hours was more pronounced during the wet season. After this, both parameters declined relatively consistently, during both seasons. Overall, *G*_*t*_ and *E*_*L*_ were consistently higher during the wet season, with the estimated *E*_*L*_ values for *L. speciosa* being twice that of the dry season. A plateau during the wet season (less pronounced in the dry season), was observed for *S. macrophylla* (from 9:00 to 13:00 LT).

**Figure 4:**
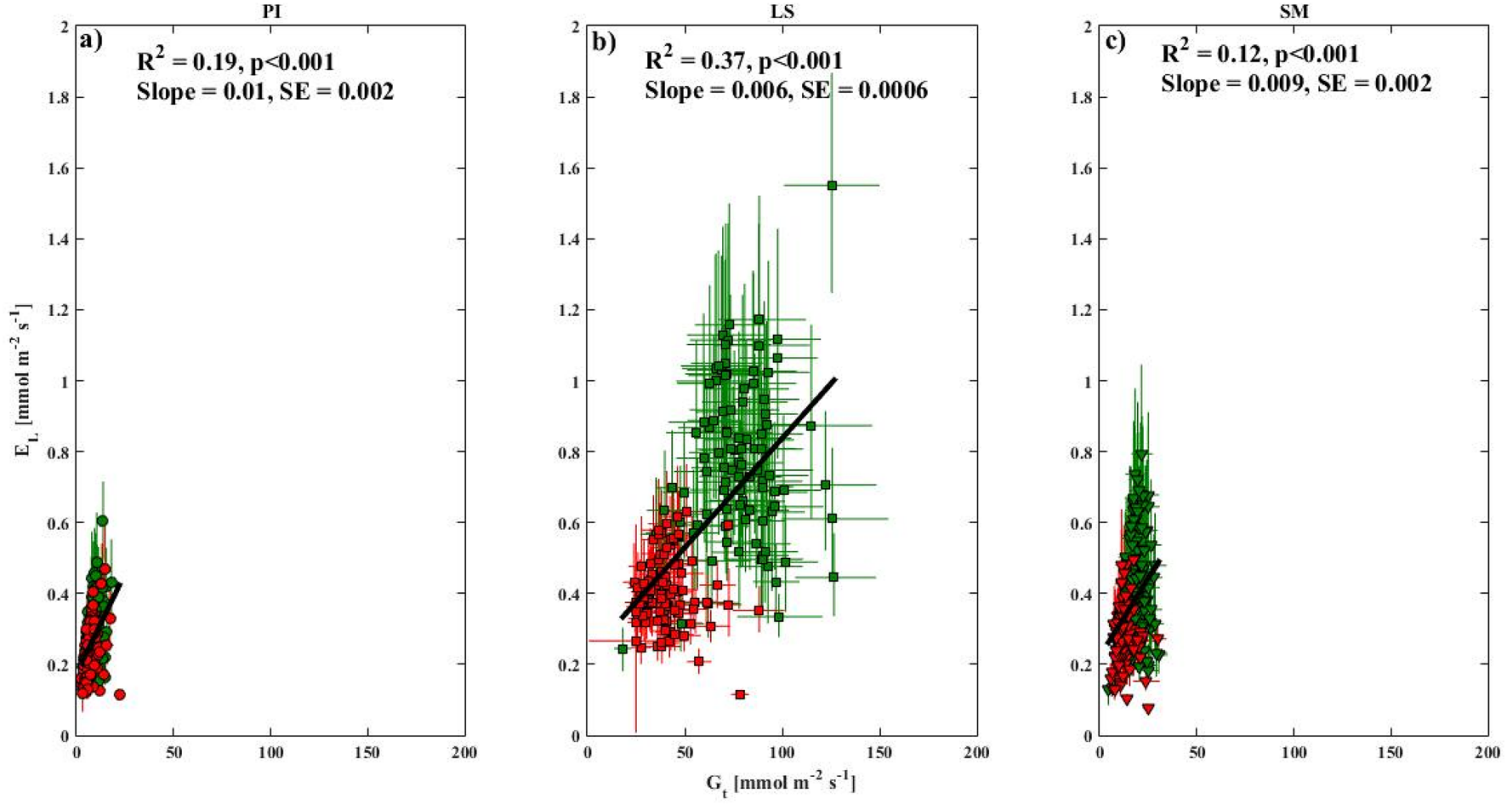
Daily averages of transpiration (*E*_*L*_) plotted against canopy conductance (*G*_*t*_) for (**a**) *P. indicus* (PI), (**b**) *L. speciosa* (LS), and (**c**) *S. macrophylla* (SM) and seasons plotted in green (wet) and red (dry) symbols. The black lines are linear fits, with the corresponding regression statistics indicated in each panel. This figure was generated using MATLAB version 7.12.0 R2017a (https://www.mathworks.com/products/new_products/release2017a.html).

Over various time scales (weekly and monthly not shown), *E*_*L*_ was positively correlated with *G*_*t*_ within both wet and dry seasons (Fig. 4 showing daily estimates only). The significant relationships revealed that, on average over both seasons, a unit change in *G*_*t*_ caused a change of 0.45, 1.16, and 0.76 units in *E*_*L*_ for *P. indicus*, *L. speciosa*, and *S. macrophylla*, respectively.

### Atmospheric and soil demand

**Figure 5:**
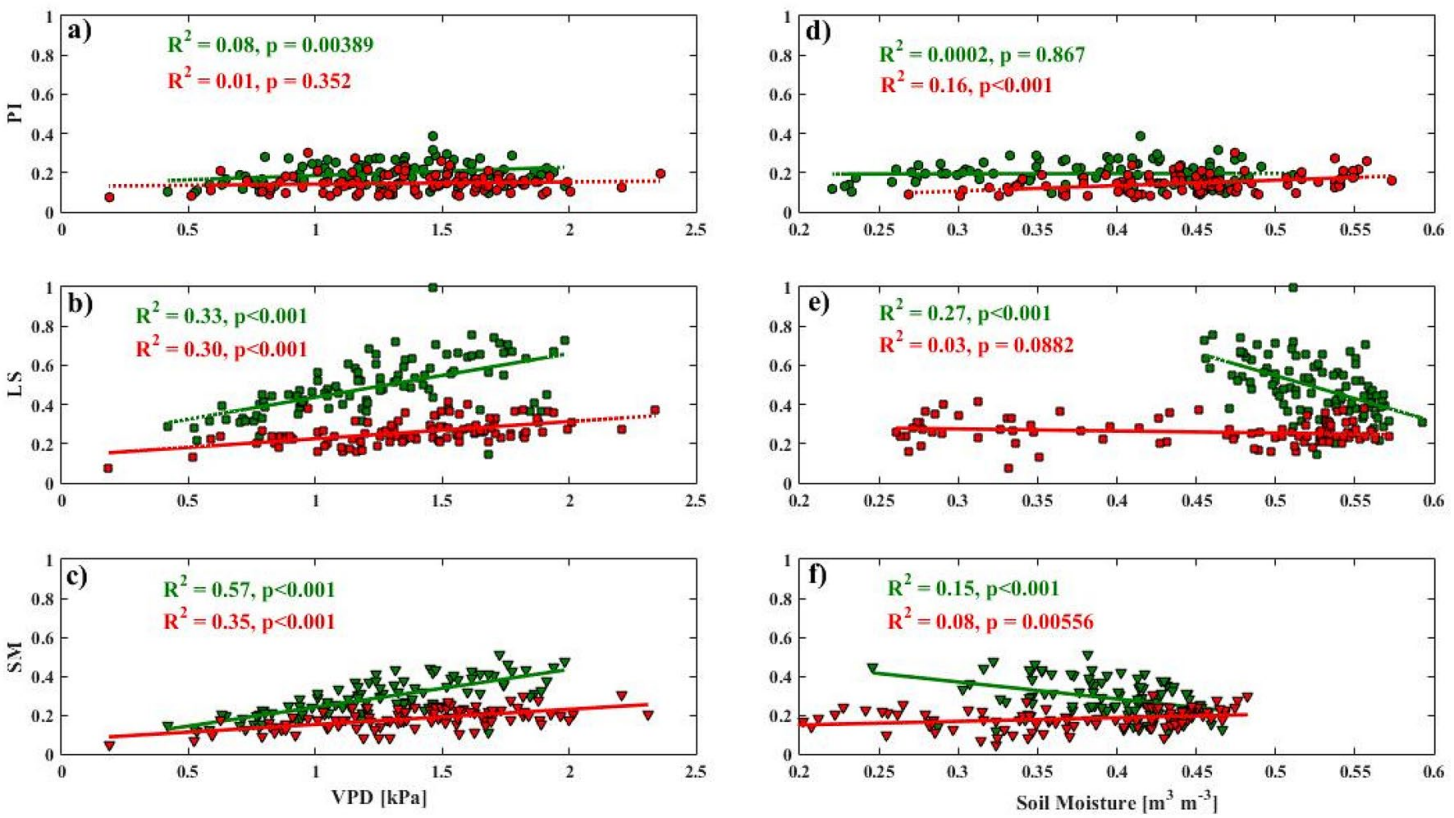
Daily averages of transpiration (*E*_*L*_) plotted against VPD (**a**–**c**) and soil moisture (**d**–**f**) with the rows corresponding to the three species: *P. indicus* (PI), *L. speciosa* (LS), and *S. macrophylla* (SM), and seasons plotted in green (wet) and red (dry) symbols. This figure was generated using MATLAB version 7.12.0 R2017a (https://www.mathworks.com/products/new_products/release2017a.html).

Responses of daily *E*_*L*_ to atmospheric evaporative demand, represented by VPD, and soil moisture, were different between the two seasons and species (Fig. 5). Generally, *E*_*L*_ increased with increasing VPD (Fig. 5a–c) in all species. The sensitivity of *E*_*L*_ to VPD was relatively higher during the wet season and highly pronounced for *L. speciosa* and *S. macrophylla* (compare green and red lines in Fig. 5b and c), while that for *P. indicus* was similar in both seasons (Fig. 5a). Strong decreases in *E*_*L*_ with increasing soil moisture was observed during the wet season for *L. speciosa* and *S. macrophylla,* while the effect was highly suppressed in *P. indicus* (green lines in Fig. 5d–f). The observed trends of decreasing *E*_*L*_ with increasing soil water indicated possible flooding condition during the wet season. Some extra rainfalls may contribute to this, although we had no access to rainfall data at the site to confirm it. However, the range of soil moisture during the wet season was relatively small with differences between the maximum and the minimum around 0.1 m^3^ m^−3^ for both species (Fig. 5e, f) and the significant trends may be driven by the ‘extreme’ value such as the data point at the lowest soil moisture of *S. macrophylla* (the leftmost green symbol in Fig. 5f). During the dry season, *E*_*L*_ did not change significantly for both *L. speciosa* and *S. macrophylla* but it increased marginally with increasing soil moisture for *P. indicus*.

## Discussion

### Model performance to predict sap flux density in saplings

Using the Bayesian modeling technique^31^, we estimated *G*_*t*_ and *E*_*L*_ from sap flux density measured in saplings of three common urban species in Bangkok. The sap flux rates were closely related to the weather variations, particularly VPD. Previous studies have indicated that the stomatal response is sensitive to VPD as it directly affects the leaf water loss which, together with plant hydraulic system, controls leaf water potential and turgor pressure^39–42^. The best model performance was obtained by uniquely parameterizing each species and season with variable dampening effects (as indicated by the *β* parameters in Table 1), based on the dependence of water storage and hence transpiration on changes in soil and plant water status^43^. Given many uncertainties related to measurement errors (e.g., those in measuring environmental data), the overall agreement between the model estimates and the data was sufficiently accurate, as indicated by the respective R^2^ and RMSE values, implying that the model estimates of *G*_*t*_ and *E*_*L*_ were reliable. Furthermore, VPD and soil moisture could explain a large portion of the variability in the sap flux density of the three species, as indicated by mean R^2^ during wet (dry) seasons; 0.64 (0.65) for *P. indicus*, 0.70 (0.57) for *L. speciosa*, and 0.81 (0.78).

While the model output gives reasonable values of sap flux density which allows good estimates of *G*_*t*_ and *E*_*L*_ across the three species, certain important caveats need to be considered. In our study, assumptions were made while predicting *J*_*t*_, *G*_*t*_, and *E*_*L*_ from the sensor data, soil moisture, and environmental variables. First, we assumed that there were no radial or azimuthal variations in sap flux density, due to the small stem size. Additionally, as the non-conducting wood (pith) was negligible, it was assumed that the sapwood covered the entire stem. Thus, the estimated values are for potted saplings and more mature individuals could have a relatively modest variation between the estimated values of *G*_*t*_ and *E*_*L*_, due to a higher stomatal sensitivity to VPD^44,45^. The difference between present study and the previous study^31^ that employed the StaCC model is that their estimations were based on sap flux density in mature trees and were closely related to the total leaf area index of the crown, without the need to individually quantify the leaves that constitute it. Also, due to many unusable data points, we did not use the PAR to generate the estimates. However, we tested the model run with the data provided by the original model^31^, including and excluding the PAR sub-model, resulting in an approximately 2% difference between the two scenarios (0.86 with PAR and 0.88 without PAR). The model setup and parameterization may thus hide errors associated with such assumptions on canopy structure and missing information through appropriate parameter values. As indicated by the R^2^ and RMSE values, the model *G*_*t*_ and *E*_*L*_ estimates could be generated without a substantial loss in model performance.

### Canopy conductance and transpiration

The distribution of *G*_*t*_ was observed to be much broader for *L. speciosa*, relative to *P. indicus* and *S. macrophylla*, and approaching maximum *G*_*t*_ during the wet season (Table 2 and Fig. 2). Species-specific stomatal response is sensitive to environmental drivers, including atmospheric and soil moisture conditions, in addition to species phenology, which may promote a greater water flux during specific periods. The observation of relatively high levels of *G*_*t*_ in *L. speciosa* (deciduous species^7^) may be attributed to the fact that drought deciduous species tend to maximize carbon gain during the growing season (wet season), at the cost of a shorter leaf longevity compared to dry evergreen species^46^. *P. indicus* and *S. macrophylla*, which are evergreen species, had relatively lower values of *G*_*t*_ and *E*_*L*_ when compared to the deciduous *L. speciosa*, during the study period. The evergreen species are able to photosynthesize all year round^47^, without losing all the leaves in the canopy during any season^48^. Therefore, opened stomata for photosynthesis can be maintained at the same rate throughout the seasons (Table 2).

During the wet period, we found that the atmospheric demand (VPD) was higher than in the dry period from 10 am to 6 pm (Fig. 3a) resulting in a higher canopy conductance and transpiration. Typically, VPD during the dry period should be higher than the wet period. However, in our experiments, saplings were placed on the roof top of a building, which could have had some confounding effect of heat dissipation from the building or the cement floor resulting in a high VPD during the wet season.

In the present study, as the saplings were potted, isolated, well ventilated, and placed on the balcony of a building, the variability in *G*_*t*_ was closely related to the atmospheric demand, with an approximate but weak linear relationship observed between daily variability in *G*_*t*_ and *E*_*L*_ (Fig. 5)^49^. During the dry season, *G*_*t*_ and *E*_*L*_ were greatly reduced in all three species, which could indicate a moderation in the internal water potential, with the most prominent decrease in *L. speciosa*. The diurnal course of *G*_*t*_ and *E*_*L*_ was also variable between the three species (Fig. 5). In a previous study^50^, it was reported that the sap flux density peaked just before noon and then decreased gradually throughout the late afternoon. This could be explained by a relatively higher stomatal closure in all species under high evaporative demand.

### Soil and atmospheric demand

Stomatal response to VPD is usually optimized by minimizing water loss for a given amount of carbon assimilation^51^. Apart from sustaining a high rate of assimilation, this adaptability also helps in maintaining optimum leaf temperatures^52^. A reduction in the available soil moisture can lead to stomatal closure, reducing the evaporative demand on the internal water potential and decreasing the rate of depletion in root zone water^53^. Similar relationships with soil moisture have been observed in the Amazonian^54^ and Bornean rain forests^55^ and may reflect a response mechanism to counteract short-term water stress induced by partial drying of root zone during dry periods.

A previous study^56^ reported that *S. macrophylla* was able to extract water at relatively lower levels of soil water content. However, our study was on potted saplings, whose root expansion was limited in a container, where *S. macrophylla* was able to extract most water in the container until the soil moisture reached 0.2 m^3^ m^−3^ in dry period (Fig. 5b). The highest transpiration rates estimated for *L. speciosa* might indicate a low xylem resistance to water flow and a deep root system^57^ to maintain the water flow during dry season. Soil moisture used by *L. speciosa* during the dry season was also the highest among the three species and was above 0.25 m^3^ m^−3^ even during times when the estimated transpiration rate was the highest. This could be due to the total leaf area of *L. speciosa* being the lowest, such that the rate of water depletion in the container was lower than that for *S. macrophylla* and *P. indicus*.

### Suitable species for restoration under a future climate change scenario

Tropical species may be susceptible to variation in temperature, with a relatively small change in mean temperature causing a disproportionately large change in plant function due to heat related stresses. Additionally, extreme weather events due to erratic precipitation patterns can lead to an increased likelihood of excessive precipitation or protracted dry spells. The adaptability of a species to respond to such occurrences would strongly impact their survival. This trade-off can cause susceptibility to drought conditions in those plants that have been growing in waterlogged conditions and vice versa^58^. Such large variations in temporal difference in soil moisture conditions may be detrimental for endangered species having narrow physiological tolerances^59^ or poor competitors^60^.

Forests degraded due to prolonged mono-crop practice, from fires, or a high mortality rate resulting from climate change, can be restored back from a state of being totally razed. Buffer zones around national parks fall under a sensitive land use criterion, and are treated as a protected area. But native people residing in such areas are allowed to live of the land while being under strict regulatory supervision. Illegal deforestation and ill-effects of climate change have resulted in a high mortality rate of forest species. One strategy for devising a restoration plan is to choose native species because the introduced alien species may easily spread and are harmful to the native species.

Deciduous species exhibit higher levels of transpiration and greater cooling and drought avoidance through leaf loss, relative to evergreen species, which have a limited physiological and morphological adaptability to survive wider swings in water and temperature ranges^61,62^. While evergreen species, mostly native to wetter environments, can have fine roots concentrated near the soil surface during the dry season as nutrients and water are readily available on the soil surface. Therefore, when water is not a limiting factor on the forest floor, evergreen species compete for nitrogen and light resources^63^, which under a restoration scenario, apart from soil water and nutrients are key limiting factors determining plant survival.

All three species in this study are native to tropical regions and can be considered for establishment under a restoration program. Moreover, since all of them are fast-growing species, they can also be pioneer species in degraded or barren lands as they would provide shade for other species. Under circumstances of low soil nutrition and the need for high rate of nutrient cycling, *L. speciosa* could be a suitable choice for restoration as it tends to photosynthesize rapidly, as indicated by higher canopy conductance and transpiration (Table 2 and Fig. 2). Furthermore, given its deciduous nature, it would shed leaves during the dry season resulting in higher return rates of soil nutrient recycling.

Under a climate change scenario with higher temperatures and drier atmosphere and soil water stress, *P. indicus* is likely to be suitable choice under the restoration program, as it maintained a steady canopy conductance and transpiration during both wet and dry season soil moisture and air vapor demand (Fig. 3). This suggests that *P. indicus* would maintain a steady growth rate, independent of how dry the soil or air is. In other words, the stomata of *P. indicus* are relatively less sensitive to air and soil moisture demands when compared to *L. speciosa* and *S. macrophylla*. Also, *P. indicus* would be able to survive even if the future climate is predicted to be wetter than at present. However, the only downside of considering *P. indicus* is its potentially relatively slow growth rate due to low stomatal opening for photosynthesis but if the annual growth is to be accounted for, it may not be much different from the other two species.

## Conclusions

We report the model estimates of *G*_*t*_ and *E*_*L*_ for the three potted sapling tree species across temporal scales during the wet and dry season using the StaCC model, which has been previously used only with the mature tree species. With our unique parameterization for species and season, we concluded that the model was able to perform well with the sapling data and could be used further to provide meaningful estimates of *G*_*t*_ and *E*_*L*_ for saplings. As the estimated transpiration rates of the studied species were variable, it is possible to select the most suitable species to be planted accordingly to restore degraded lands for effective reforestation. Transpiration per unit leaf area was the highest in *L. speciosa* while that in *S. macrophylla* and *P. indicus* was lower in both seasons. During the wet season with high VPD, higher amount of water loss through transpiration occurred compared to the dry season with a lower VPD. *P. indicus* is likely to be insensitive to changes of soil moisture and atmospheric demands, while *L. speciosa* responded to even small variations in the weather conditions. Leaf phenology might play a crucial role in *G*_*t*_ and *E*_*L*_ due to differences in the species’ behavior. Being a deciduous species, *L. speciosa* would have a stronger stomatal control, while *P. indicus* and *S. macrophylla*, as evergreen species, tended to have low water use, given the ability to photosynthesize all year round, and would be able to maintain their stomatal opening even under the high atmospheric demand. Nevertheless, future studies on field-grown trees should be performed to confirm the findings.

## Methods

### Experimental setup and environmental measurement

The study site was established on the balcony of the 4th floor of a building in Chulalongkorn University, Bangkok (13° 44′ 2.9′′ N 100° 31′ 54.1′′ E). Based on the 30-year- record (1981–2010) of climate data from a Bangkok metropolis station (Thai Meteorological Department), mean annual air temperature was 28.6 °C and mean annual precipitation was 1648 mm. This study was undertaken from 25th July 2017 to 11th February 2018, which included both the wet (25th July–31st October 2017) and dry season (1st November 2017 to 11th February 2018). Ten trees for each species were bought from a nursery (Chatuchak market) and an automatic irrigation system was used to water the trees once a day. Despite the daily irrigation, the fluctuations of soil moisture may be large enough to affect any physiological responses of the potted saplings, hence we considered soil moisture as a covariate in this study. A detailed description of the experimental setup and the measurements can be found in a previous study^50^ which mainly reported the observed sap flux density without scaling up to canopy conductance. We measured half-hourly air temperature (°C) and relative humidity (RH in %) with a probe (HC23-L, Campbell Scientific, Logan UT, USA), which were used to calculate VPD (kPa)^64^. Photosynthetically active radiation (PAR, umol m^−2^ s^−1^) was measured using a quantum sensor (LI190R-L, Li-COR Biosciences, Lincoln, NE, USA. All these instruments were installed at approximately 2 m above the saplings. Volumetric soil moisture (% by volume) was monitored using Time-Domain Reflectometry (TDR) probes (CS 616, Campbell Scientific, Logan UT, USA), with the default factory calibration, installed in 2 pots for *L. speciosa* and *S. macrophylla* and 3 pots for *P. indicus*.

### Tree biometric data

Tree data, including girth at breast height (GBH in cm), and leaf area were also measured to parameterize the model. The GBH was converted to diameter at breast height (DBH), assuming the stems were circular. Sapwood area was estimated from the measured GBH using the equation for calculating a circle, assuming negligible non-conducting wood (pith) due to small stem size (GBH ranged 7.3–10.7 cm). The assumption of negligible pith was confirmed from cutting some stems in the previous experiment and found that 98% of the basal area was sapwood area. For leaf area, we sampled 5 leaves from the upper and lower half of the crown of each sapling, scanned using a scanner (EPSON L3110 model) and analyzed the areas using ImageJ^65^. Total leaf area of each sapling was then determined by multiplying the average measured leaf areas with the number of leaves in the respective crown layer. For scaling purposes using the model, leaf area and sapwood area were determined at the beginning of the study. We realized that leaf shedding may occur in some species, which could change the leaf areas of the studied saplings. However, since the study period was of 6 months, we could not get significant change in leaf area. Also, we considered to preserve the leaves because collecting them for the measurement may influence the sap flux measurements through changing transpirational area. Mean values (± SD) of total leaf area of the saplings were 3 ± 0.34 m^2^ for *P. indicus*, 2.55 ± 0.18 m^2^ for *L. speciosa* and 2.97 ± 0.28 m^2^ for *S. macrophylla*.

### Sap flux measurement

To measure sap flux density (*J*_*t*_; g m^−2^ s^−1^), of the saplings, Granier-type thermal dissipation probes^20,21^ (TDPs) were constructed. Each T-type thermocouple (copper-constantan) was encapsulated within a steel needle making up the probe. A data logger (used to measure temperature difference between the two probes) was connected to each copper end with the constantan ends connected to each. The sap flux rate was measured continuously by tracking the difference between the unheated (upstream or lower probe) and the heated (downstream or upper probe). The temperature difference was converted to sap flux density using the Baseliner program version 4.0^66^. All environmental sensors and sap flux probes were connected to a datalogger (CR1000, Campbell Scientific, Logan, UT, USA) which recorded 30-min average data throughout the study period. However, due to sensor failure for a relatively long time, we could not obtain continuous PAR record, with gaps covering 39% of the period. Consequently, the PAR data were not considered in further analysis after finding only 2% difference between running the model with and without the PAR sub-model.

### Model description

We used the State-Space Canopy Conductance (StaCC) model to gap-fill the missing sap flow measurements using Bayesian inference, and to estimate the latent variables such as canopy stomatal conductance (*G*_*t*_), transpiration per unit leaf area (*E*_*L*_), and canopy transpiration (*E*_*c*_)^31^. The model utilizes a combination of sap flux data and canopy conductance models while accounting for random errors associated with individual sensors. The final hierarchical Bayesian construct is a joint distribution accounting for sap flux measurement, latent states (transpiration and canopy conductance), and model parameters. The data model uses the measured sap flux density to estimate sap flux rate in probe *i* at time *t* as:

1 $$J_{it} \sim {\text{ N}}\left( {J_{t} {\text{Z}}\left( {d_{i} } \right)a_{i} ,S} \right)$$

where *J*_*t*_ is the average sap flux at time *t*, Z(*d*_*i*_) is the sapwood depth sub-model, *a*_*i*_ is the random effect due to probe *i* and *S* is the observational variance. As the sap flux was measured on potted saplings with small stem size (GBH ~ 7.3–10.7 cm), the sapwood depth sub-model was not used in this study. The model estimates canopy conductance (*G*_*t*_) and transpiration per unit leaf area (*E*_L_). The steady-state conductance at instant *t* (*G*_*s,t*_*,* mmol m^−2^ s^−1^) is modeled semi-mechanistically^67,68^ through multiplicative nonlinear functions of environmental covariates, including vapor pressure deficit (*D*_*t*_), photosynthetically active radiation (*Q*_*t*_), and volumetric soil moisture (*M*_*t*_) as:

2 $$G_{s,t} = {\text{ f}}\left( {D_{t} } \right){\text{g}}\left( {Q_{t} } \right){\text{h}}\left( {M_{t} } \right)$$

The sub-model estimating the effect of *D*_*t*_ on *G*_*s,t*_ has the form:

3 $${\text{f}}\left( {D_{t} } \right) \, = G_{ref} {-} \, \lambda {\text{ ln}}\left( {D_{t} } \right)$$

where *G*_*ref*_ is the reference conductance (or the canopy conductance at *D*_*t*_ = 1 kPa) and λ is the stomatal sensitivity to *D*_*t*_^68^. The dependence of *G*_*s,t*_ on light is incorporated through the sub-model:

4 $${\text{g}}\left( {Qt} \right) \, = { 1 }{-} \, \alpha_{{1}} {\text{exp}}\left( {Q_{t} /\alpha_{{2}} } \right)$$

where, α_1_ accounts for nighttime conductance and α_2_ is the sensitivity to *Q*_*t*_. However, because of the large gaps in PAR record, we did not use this light sub-model, as explained previously. The soil moisture sub-model has the form:

5 $$\begin{aligned} & {\text{h}}\left( {M_{t} } \right) \, = {\text{ exp }}( - 0.{5}\left( {M_{t} {-} \, \alpha_{{3}} } \right)^{{2}} /\alpha_{{4}}^{{2}} \quad {\text{if}}\;M_{t} \le \, \alpha_{{3}} \\ & {\text{or}}\;{\text{ h}}\left( {M_{t} } \right) \, = {\text{ 1 if}}M_{t} > \, \alpha_{{3}} , \\ \end{aligned}$$

where α_3_ is the threshold below which *M*_*t*_ reduces *G*_*s,t*_ and α_4_ controls the sensitivity of the reduction in *G*_*s,t*_ with decrease in *M*_*t*_ below threshold. *G*_*s,t*_ is used to calculate actual canopy conductance (*G*_*t*_) using the state equation:

6 $$G_{t} = G_{t - dt} + \, \left( {G_{s,t} {-}G_{t - dt} } \right)V_{t} ,$$

with the assumption that *G*_*t*_ depends on the conductance at previous time step *t−dt* and *dt* = 30 min and *V*_*t*_ = 1 − exp(−*dt*/τ). The *V*_*t*_ term accounts for stomatal lags and τ = 10 min^31^. Canopy conductance is then scaled to calculate *E*_*L*_ (kg m^−2^ s^−1^) as:

7 $$E_{L,t} = G_{t} q_{t}$$

where *q*_*t*_ is a composite variable. We kept a weak prior on the species-specific lags in the capacitance sub-model described in the previous study^31^. Additionally, wherever possible, the same priors for the data and process models were used^31^. A separate model analysis was implemented for each of the three species and the two seasons. The Gibbs sampler was run for 10,000 iterations and the first 3000 iterations were discarded as burn-in. The model generated *J*_*t*_, *G*_*t*_, and *E*_*L*_ estimates at each time step and was used to evaluate the differences in water flux among species and across the wet and dry seasons. Daily, weekly, and monthly averages of *J*_*t*_, *G*_*t*_, and *E*_*L*_, were calculated for each species to evaluate the variability in water flux across temporal scales. We then estimated the relationship between daily *G*_*t*_ and *E*_*L*_ and environmental variables (VPD and soil moisture) using linear regression analyses. The analyses and visualization were carried out on MATLAB 7.12.0 R2017a (The MathWorks, Inc., Natick, Massachusetts, USA). To determine the interspecific and inter-seasonal differences, ANOVA and multiple regressions associated with mean parameters were performed, using the MATLAB program.

## Data Availability

The datasets generated during and/or analyzed during the current study are available from the corresponding author on reasonable request.

## References

[1] Pan Y (2011). A large and persistent carbon sink in the world’s forests. Science.

[2] Blenkinsop S, Fowler HJ (2007). Changes in drought frequency, severity and duration for the British Isles projected by the PRUDENCE regional climate models. J. Hydrol..

[3] Guardiola-Claramonte M (2011). Decreased streamflow in semi-arid basins following drought-induced tree die-off: a counter-intuitive and indirect climate impact on hydrology. J. Hydrol..

[4] Hartmann H (2018). Research frontiers for improving our understanding of drought-induced tree and forest mortality. New Phytol..

[5] Chaturvedi RK, Raghubanshi AS, Tomlinson K, Singh JS (2017). Impacts of human disturbance in tropical dry forests increase with soil moisture stress. J. Veg. Sci..

[6] Sjöman H, Hirons AD, Bassuk NL (2018). Improving confidence in tree species selection for challenging urban sites: a role for leaf turgor loss. Urban Ecosyst..

[7] Esperon-Rodriguez M (2019). Assessing the vulnerability of Australia’s urban forests to climate extremes. Plants People Planet..

[8] Thaiutsa B, Puangchit L, Kjelgren R, Arunpraparut W (2008). Urban green space, street tree and heritage large tree assessment in Bangkok, Thailand. Urban For. Urban Green..

[9] Chaturvedi, R.K., Tripathi, A., Raghubanshi, A.S. & Singh, J.S. Functional traits indicate a continuum of treee drought strategies across a soil water availability gradient in a tropical dry forest. *For. Ecol. Manag.* 2020 (In press).

[10] Chaturvedi RK, Raghubanshi AS, Singh JS (2013). Growth of tree seedlings in a tropical dry forest in relation to soil moisture and leaf traits. J. Plant Ecol..

[11] Krauss KW, Twilley RR, Doyle TW, Gardiner ES (2006). Leaf gas exchange characteristics of three neotropical mangrove species in response to varying hydroperiod. Tree Physiol..

[12] Yan M-J, Yamanaka N, Yamamoto F, Du S (2010). Responses of leaf gas exchange, water relations, and water consumption in seedlings of four semiarid tree species to soil drying. Acta Physiol. Plant..

[13] Yan W, Zheng S, Zhong Y, Shangguan Z (2017). Contrasting dynamics of leaf potential and gas exchange during progressive drought cycles and recovery in *Amorpha fruticosa* and *Robinia pseudoacacia*. Sci. Rep..

[14] Medlyn BE (2001). Stomatal conductance of forest species after long-term exposure to elevated CO_2_ concentration: a synthesis. New Phytol..

[15] Wullschleger SD, Gunderson CA, Hanson PJ, Wilson KB, Norby RJ (2002). Sensitivity of stomatal and canopy conductance to elevated CO_2_ concentration: interacting variables and perspectives of scale. New Phytol..

[16] Ainsworth EA, Rogers A (2007). The response of photosynthesis and stomatal conductance to rising [CO_2_]: mechanisms and environmental interactions. Plant. Cell Environ..

[17] Tor-ngern P (2015). Increases in atmospheric CO_2_ have little influence on transpiration of a temperate forest canopy. New Phytol..

[18] Schäfer KVR (2003). Exposure to an enriched CO_2_ atmosphere alters carbon assimilation and allocation in a pine forest ecosystem. Glob. Chang. Biol..

[19] Williams M (1996). Modelling the soil-plant-atmosphere continuum in a Quercus-Acer stand at Harvard Forest: the regulation of stomatal conductance by light, nitrogen and soil/plant hydraulic properties. Plant. Cell Environ..

[20] Granier A (1985). Une nouvelle méthode pour la mesure du flux de sève brute dans le tronc des arbres. Ann. For. Sci..

[21] Granier A (1987). Evaluation of transpiration in a Douglas-fir stand by means of sap flow measurements. Tree Physiol..

[22] Green S, Clothier B, Jardine B (2003). Theory and practical application of heat pulse to measure sap flow. Agron. J..

[23] Burgess SSO (2001). An improved heat pulse method to measure low and reverse rates of sap flow in woody plants. Tree Physiol..

[24] Sakuratani T (1981). A Heat balance method for measuring water flux in the stem of intact plants. J. Agric. Meteorol..

[25] Chang X, Zhao W, Zhang Z, Su Y (2006). Sap flow and tree conductance of shelter-belt in arid region of China. Agric. For. Meteorol..

[26] Ewers BE, Oren R, Phillips N, Strömgren M, Linder S (2001). Mean canopy stomatal conductance responses to water and nutrient availabilities in *Picea abies* and *Pinus taeda*. Tree Physiol..

[27] Pataki DE, Oren R, Phillips N (1998). Responses of sap flux and stomatal conductance of *Pinus taeda* L. trees to stepwise reductions in leaf area. J. Exp. Bot..

[28] Ryan MG (2000). Transpiration and whole-tree conductance in ponderosa pine trees of different heights. Oecologia.

[29] Oishi AC, Oren R, Novick KA, Palmroth S, Katul GG (2010). Interannual invariability of forest evapotranspiration and its consequence to water flow downstream. Ecosystems.

[30] Oishi AC, Oren R, Stoy PC (2008). Estimating components of forest evapotranspiration: a footprint approach for scaling sap flux measurements. Agric. For. Meteorol..

[31] Bell DM (2015). A state-space modeling approach to estimating canopy conductance and associated uncertainties from sap flux density data. Tree Physiol..

[32] Kim H-S, Oren R, Hinckley TM (2008). Actual and potential transpiration and carbon assimilation in an irrigated poplar plantation. Tree Physiol..

[33] Meinzer FC, James SA, Goldstein G (2004). Dynamics of transpiration, sap flow and use of stored water in tropical forest canopy trees. Tree Physiol..

[34] Phillips N, Nagchaudhuri A, Oren R, Katul G (1997). Time constant for water transport in loblolly pine trees estimated from time series of evaporative demand and stem sapflow. Trees.

[35] Tor-ngern P (2017). Ecophysiological variation of transpiration of pine forests: synthesis of new and published results. Ecol. Appl..

[36] Clark JS (2011). Inferential ecosystem models, from network data to prediction. Ecol. Appl..

[37] Lu P, Urban L, Zhao P (2004). Granier’s thermal dissipation probe (TDP) method for measuring sap flow in trees: theory and practice. Acta Bot. Sin..

[38] Ewers B, Oren R (2000). Analyses of assumptions and errors in the calculation of stomatal conductance from sap flux measurements. Tree Physiol..

[39] Ward EJ, Oren R, Sigurdsson BD, Jarvis PG, Linder S (2008). Fertilization effects on mean stomatal conductance are mediated through changes in the hydraulic attributes of mature Norway spruce trees. Tree Physiol..

[40] Addington RN, Mitchell RJ, Oren R, Donovan LA (2004). Stomatal sensitivity to vapor pressure deficit and its relationship to hydraulic conductance in *Pinus palustris*. Tree Physiol..

[41] Leuning R (1995). A critical appraisal of a combined stomatal-photosynthesis model for C3 plants. Plant. Cell Environ..

[42] Meinzer FC, Hinckley TM, Ceulemans R (1997). Apparent responses of stomata to transpiration and humidity in a hybrid poplar canopy. Plant. Cell Environ..

[43] Monteith JL (1995). A reinterpretation of stomatal responses to humidity. Plant. Cell Environ..

[44] Loustau D (1996). Transpiration of a 64-year-old maritime pine stand in Portugal. Oecologia.

[45] Domec J-C, Gartner BL (2001). Cavitation and water storage capacity in bole xylem segments of mature and young Douglas-fir trees. Trees.

[46] Moore GW, Bond BJ, Jones JA, Phillips N, Meinzer FC (2004). Structural and compositional controls on transpiration in 40- and 450-year-old riparian forests in western Oregon, USA. Tree Physiol..

[47] Leigh A, Sevanto S, Close JD, Nicotra AB (2017). The influence of leaf size and shape on leaf thermal dynamics: does theory hold up under natural conditions?. Plant. Cell Environ..

[48] Brodribb TJ, Holbrook NM, Edwards EJ, Gutiérrez MV (2003). Relations between stomatal closure, leaf turgor and xylem vulnerability in eight tropical dry forest trees. Plant. Cell Environ..

[49] Choat B, Ball M, Luly J, Donnelly C, Holtum J (2006). Seasonal patterns of leaf gas exchange and water relations in dry rain forest trees of contrasting leaf phenology. Tree Physiol..

[50] Tor-ngern P, Puangchit L (2018). Effects of varying soil and atmospheric water deficit on water use characteristics of tropical street tree species. Urban For. Urban Green..

[51] Jarvis PG, Cannel MGR, Jackson JE (1985). Transpiration and assimilation of tree and agricultural crops: the omega factor. Attributes of Trees as Crop Plants.

[52] Marchin RM, Broadhead AA, Bostic LE, Dunn RR, Hoffmann WA (2016). Stomatal acclimation to vapour pressure deficit doubles transpiration of small tree seedlings with warming. Plant. Cell Environ..

[53] Mahan JR, Upchurch DR (1988). Maintenance of constant leaf temperature by plants—I Hypothesis-limited homeothermy. Environ. Exp. Bot..

[54] Schultz HR (2003). Differences in hydraulic architecture account for near-isohydric and anisohydric behaviour of two field-grown *Vitis vinifera* L. cultivars during drought. Plant. Cell Environ..

[55] Harris PP, Huntingford C, Cox PM, Gash JHC, Malhi Y (2004). Effect of soil moisture on canopy conductance of Amazonian rainforest. Agric. For. Meteorol..

[56] Kjelgren R, Joyce D, Doley D (2013). Subtropical-tropical urban tree water relations and drought stress response strategies. Arboric. Urban For..

[57] West AG, Hultine KR, Jackson TL, Ehleringer JR (2007). Differential summer water use by *Pinus edulis* and *Juniperus osteosperma* reflects contrasting hydraulic characteristics. Tree Physiol..

[58] Hatfield JL, Prueger JH (2015). Temperature extremes: effect on plant growth and development. Weather Clim. Extrem..

[59] Suralta RR, Yamauchi A (2008). Root growth, aerenchyma development, and oxygen transport in rice genotypes subjected to drought and waterlogging. Environ. Exp. Bot..

[60] Lawler JJ (2002). The scope and treatment of threats in endangered species recovery plans. Ecol. Appl..

[61] Liu H, Lin J, Zhang M, Lin Z, Wen T (2008). Extinction of poorest competitors and temporal heterogeneity of habitat destruction. Ecol. Modell..

[62] Baltzer JL, Grégoire DM, Bunyavejchewin S, Noor NSM, Davies SJ (2009). Coordination of foliar and wood anatomical traits contributes to tropical tree distributions and productivity along the Malay-Thai Peninsula. Am. J. Bot..

[63] Kursar TA (2009). Tolerance to low leaf water status of tropical tree seedlings is related to drought performance and distribution. Funct. Ecol..

[64] Monteith JL, Unsworth MH (1990). Principles of Environmental Physics.

[65] Schneider CA, Rasband WS, Eliceiri KW (2012). NIH Image to ImageJ: 25 years of image analysis. Nat. Methods..

[66] Oishi AC, Hawthorne DA, Oren R (2016). Baseliner: an open-source, interactive tool for processing sap flux data from thermal dissipation probes. Software X..

[67] Ball J, Woodrow I, Berry J (1987). A model predicting stomatal conductance and its contribution to the control of photosynthesis under different environmental conditions. Prog. Photosynth. Res..

[68] Jarvis PG (1976). The interpretation of the variations in leaf water potential and stomatal conductance found in canopies in the field. Philos. Trans. R. Soc. London. B Biol. Sci..

